# Fluidization and Resolidification of the Human Bladder Smooth Muscle Cell in Response to Transient Stretch

**DOI:** 10.1371/journal.pone.0012035

**Published:** 2010-08-06

**Authors:** Cheng Chen, Ramaswamy Krishnan, Enhua Zhou, Aruna Ramachandran, Dhananjay Tambe, Kavitha Rajendran, Rosalyn M. Adam, Linhong Deng, Jeffrey J. Fredberg

**Affiliations:** 1 Key Laboratory of Biorheological Science and Technology, Ministry of Education, Bioengineering College, Chongqing University, Chongqing, China; 2 Program in Molecular and Integrative Physiological Sciences, Department of Environmental Health, Harvard School of Public Health, Boston, Massachusetts, United States of America; 3 Urological Diseases Research Center, Department of Urology, Children's Hospital Boston and Department of Surgery, Harvard Medical School, Boston, Massachusetts, United States of America; Clarkson University, United States of America

## Abstract

**Background:**

Cells resident in certain hollow organs are subjected routinely to large transient stretches, including every adherent cell resident in lungs, heart, great vessels, gut, and bladder. We have shown recently that in response to a transient stretch the adherent eukaryotic cell promptly fluidizes and then gradually resolidifies, but mechanism is not yet understood.

**Principal Findings:**

In the isolated human bladder smooth muscle cell, here we applied a 10% transient stretch while measuring cell traction forces, elastic modulus, F-actin imaging and the F-actin/G-actin ratio. Immediately after a transient stretch, F-actin levels and cell stiffness were lower by about 50%, and traction forces were lower by about 70%, both indicative of prompt fluidization. Within 5min, F-actin levels recovered completely, cell stiffness recovered by about 90%, and traction forces recovered by about 60%, all indicative of resolidification. The extent of the fluidization response was uninfluenced by a variety of signaling inhibitors, and, surprisingly, was localized to the unstretch phase of the stretch-unstretch maneuver in a manner suggestive of cytoskeletal catch bonds. When we applied an “unstretch-restretch” (transient compression), rather than a “stretch-unstretch” (transient stretch), the cell did not fluidize and the actin network did not depolymerize.

**Conclusions:**

Taken together, these results implicate extremely rapid actin disassembly in the fluidization response, and slow actin reassembly in the resolidification response. In the bladder smooth muscle cell, the fluidization response to transient stretch occurs not through signaling pathways, but rather through release of increased tensile forces that drive acute disassociation of actin.

## Introduction

Cells *in vivo* are routinely subjected to mechanical stimuli that markedly influence their structure and function [1]–[5]. We have shown recently that in response to a transient stretch-unstretch maneuver, cells across a wide range of physiological systems including airway, kidney, and blood vessels, respond by promptly ablating their stiffness and cell traction forces, while transiently increasing their loss tangent [6], [7]. Taken together, these mechanical responses demonstrate that the cell acutely fluidizes [7]. These measurements also show that this fluidization response is prompt, and mediated by the effects of physical forces acting directly upon a material – the cytoskeleton – that is innately fragile [8]–[11]. However, the mechanism accounting for the fluidization phenomenon remains unclear.

The primary goal of this study is to investigate in the human bladder smooth muscle (HBSM) cell the structural and molecular level changes that underlie the fluidization response. We used imaging and molecular probes to measure dynamics of F-actin polymerization, we used Cell Mapping Rheometry (CMR) [6] to measure cell traction force dynamics, and we used Optical Magnetic Twisting Cytometry (OMTC) [12] to measure cell stiffness dynamics. To investigate specificity of the fluidization response, we pretreated HBSM cells with a panel of signaling inhibitors whose effects on bladder smooth muscle cell physiology are well known [13]–[15]. Finally, we applied to the cell instead of a transient stretch maneuver (i.e. stretch-unstretch), a transient compression maneuver (i.e. unstretch-restretch).

Although it has been shown previously that transient stretch-unstretch fluidizes the cytoskeleton (CSK), these prompt mechanical effects have been attributed entirely to the disruption of actin-myosin crosslinks and other weak stress-bearing bonds [6], [7], [16]–[21]. Depolymerization of F-actin filaments in response to stretch has also been known for a long time [22], but based upon existing data, that depolymerization process was thought to be too slow to account for prompt CSK fluidization. Here for the first time we unify these structural and mechanical changes.

## Results

### Changes of traction force

**Figure 1:**
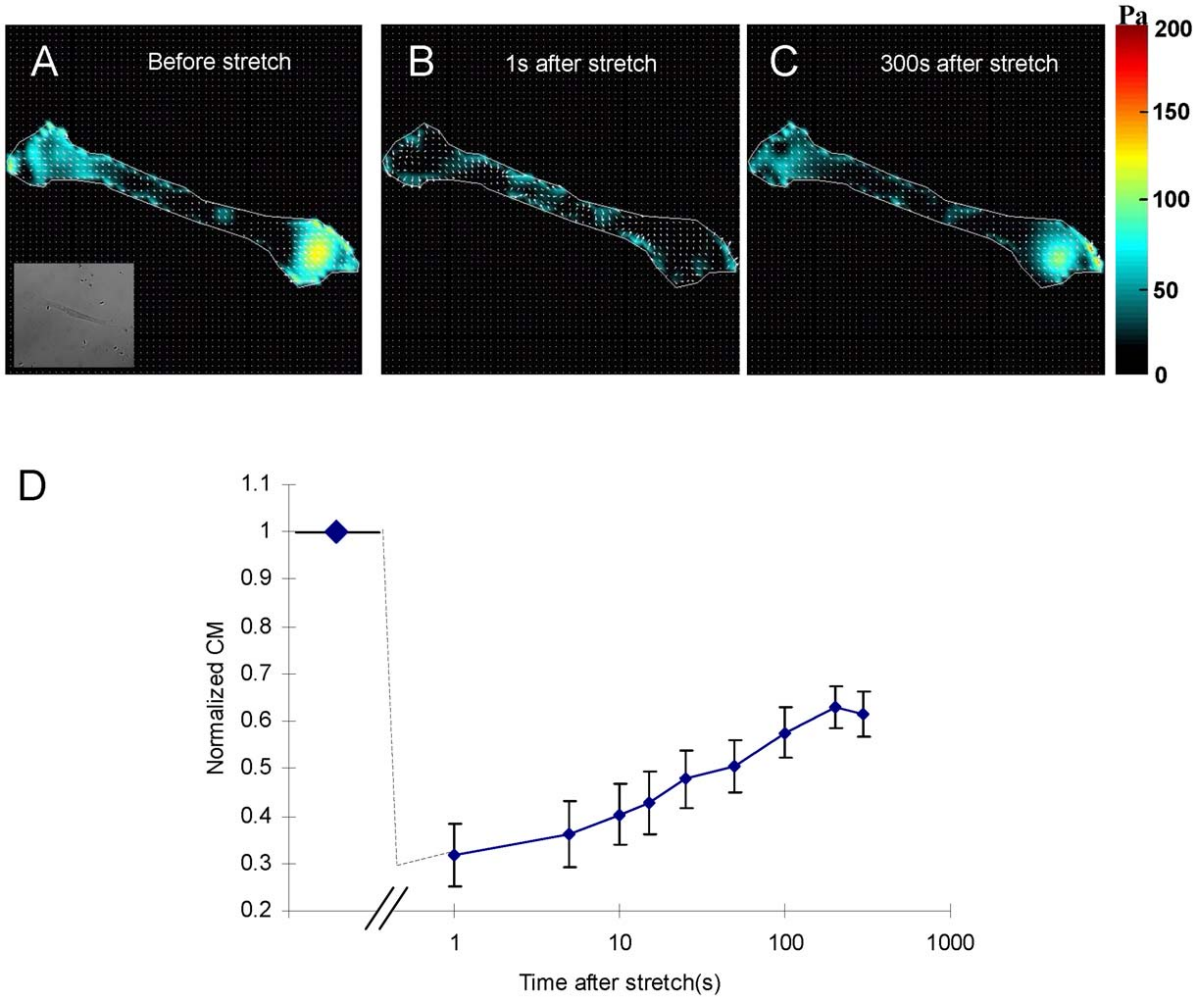
Traction force dynamics in HBSM cells subjected to a transient stretch. (A) Cell traction map before stretch. (B) Traction map immediately after a 10% homogeneous biaxial tensile stretch of a 4s duration. The cell tractions are markedly ablated. (C) Traction map measured at 300s following stress cessation. Tractions have largely recovered to the baseline value in (A). (D) Contractile moment computed from the traction maps. After transient stretch, the contractile moment decreased by 70% and then recovered to 60% of baseline value in 300s. Data are reported as mean
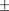
SE (n = 9 cells).

The traction force is the net force per unit area transmitted from the adherent cell to the substrate, and must be balanced by the internal stress (prestress) in the cell body [23]. After completion of a transient stretch-unstretch maneuver of 4 s duration, there was a dramatic and prompt decrease in the traction forces (Figure 1B). Within 5 min, the traction forces gradually recovered (Figure 1C) to prestretch levels (Figure 1A). The traction force dynamics were even clearer when we quantified them through the contractile moment [24]: at the earliest measurable time point following stretch, the contractile moment was reduced by 70% of its baseline value. This was followed by a gradual recovery (Figure 1D).

### Changes of cell stiffness

**Figure 2:**
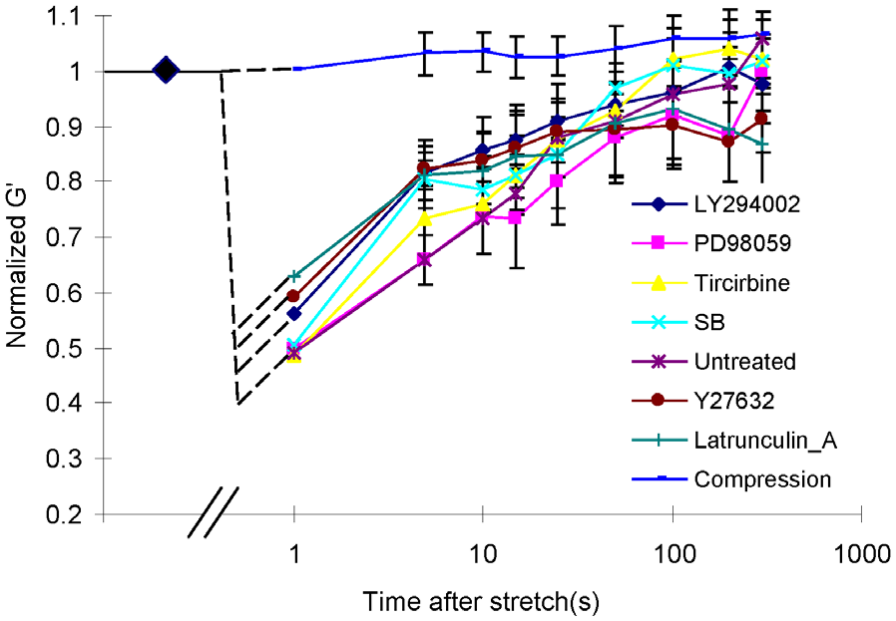
Stiffness dynamics in HBSM cells subjected to a transient stretch or a transient compression. In response to a transient tensile stretch, the elastic modulus G' dropped acutely to 50–60% of its prestretch value, then recovered slowly. The rate and the extent of this fluidization-resolidification is unaffected by the addition of LY294002, Triciribine, SB2035580, PD098059, Y27632, or Latrunculin-A (p≫0.05, Kruskal-Wallis one-way analysis). When we applied to the cell a transient compression instead of a transient stretch, G' was unchanged. All results are reported as median
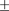
SE (n = 244–709 beads).

Stiffness after transient stretch-unstretch relative to stiffness of the same cells immediately before was denoted normalized 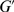 (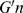). Using this normalized cell stiffness, we could use each cell as its own control. When no stretch was applied, this fractional stiffness did not change, but after cessation of a single transient stretch 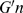 promptly decreased and then slowly recovered (Figure 2). 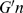 decreased to ∼50% of pre-stretch value at 5 s after stretch and returned to baseline values in 5 min (Figure 2 Untreated). Next, we tested the effects of cell signaling inhibitors that are known to play a significant role in HBSM cell mechanotransduction during a prolonged stretch and hold maneuver [13]–[15]. Contrary to their demonstrated effects during stretch and hold, these inhibitors were found to have negligible effects on cell mechanical responses following a single transient stretch-unstretch maneuver (Figure 2). When we measured HBSM cell responses following a 10% transient compression rather than a 10% transient stretch, 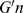 did not change (Figure 2 Compression).

### F-actin staining and levels of F-actin/G-actin

**Figure 3:**
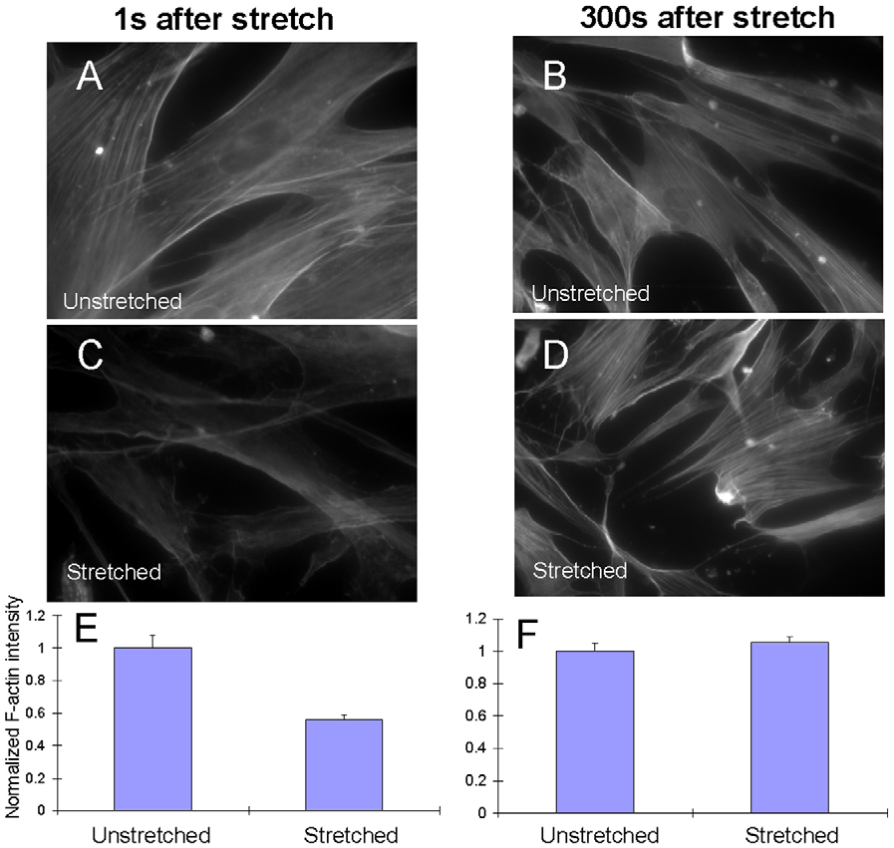
A major component of fluidization is F-actin disassembly: staining results. Within each well, F-actin levels in stretched HBSM cells (inside the indenter footprint) were compared to those in unstretched cells (outside the indenter footprint). (A),(C), F-actin filaments rapidly dissociated immediately after stretch. (B),(D), the F-actin levels recovered to baseline 300 seconds after stretch. (E),(F), from those images, we quantified intensities. Within each dish, we normalized average F-actin intensities in stretched cells to those in unstretched cells (6 dishes in total for each condition) and pooled the values together. Average F-actin intensity in stretched cells was 50% lower than that in unstretched cells immediately after stretch (Figure 3E; p<0.05, two-tailed unpaired Student's t-test), but recovered to prestretched values 300s after stretch (Figure 3F). Data are reported as mean
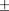
SE (n = 14–29 cells).

**Figure 4:**
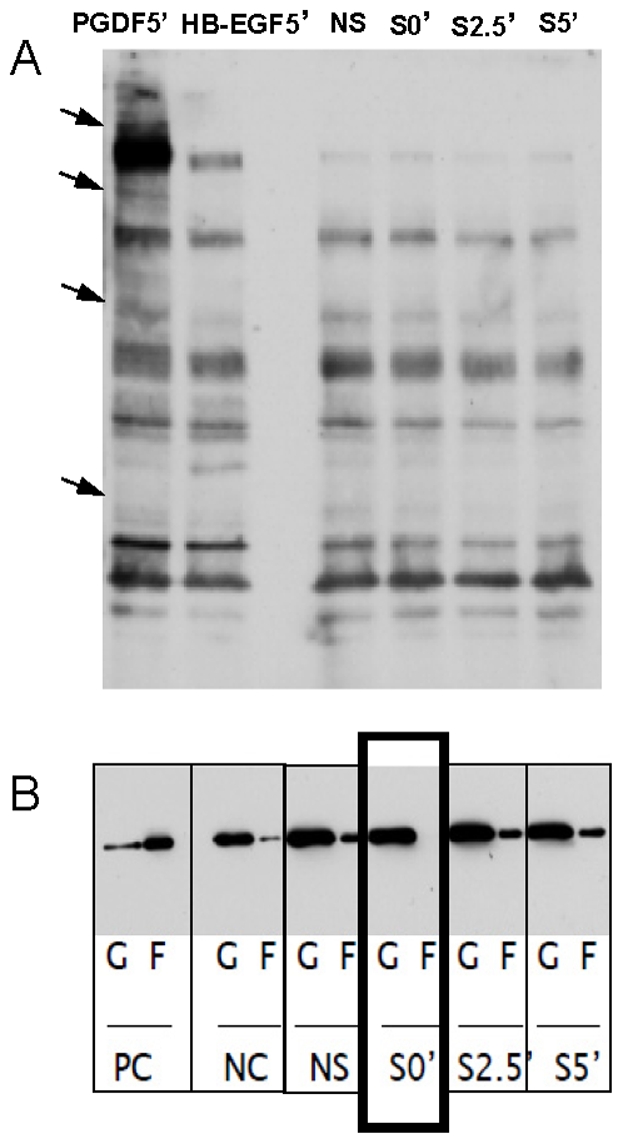
A major component of fluidization is F-actin disassembly: molecular blotting. (A) A transient stretch did not affect phosphotyrosine protein phosphorylation profiles. Positive control: PDGF and HB-EGF treatment for 5 minutes. (B) In contrast, a transient stretch acutely ablated F-actin levels at short times (S'0 – immediately after stretch). These levels recovered to baseline at longer times (S'2.5–2.5 minutes after stretch; S'5–5 minutes after stretch). Positive control (PC - 1µM phalliodin), negative control (NC - 10µM cytochalasin).

Figure 3 illustrates the representative effects of a single 10% stretch-unstretch on F-actin filaments. Compared to F-actin filaments in unstretched cells within the same dish but outside the stretched region (Figure 3A), there was clear evidence of disruption of F-actin filaments immediately following stretch (Figure 3C). Five minutes after stretch, F-actin filaments levels recovered (Figure 3D) and were indistinguishable from those in cells that were not stretched (Figure 3B). Quantitative image analysis supported this observation (Figure 3E, F). To further confirm these effects, we measured the phosphotyrosine protein phosphorylation profile and levels of filamentous (F) and globular (G) actin in HBSM cells subjected to transient stretch; over the short duration of the experimental maneuver, the total amount of actin must be conserved. The profile of phosphotyrosine protein phosphorylation did not change appreciably(Figure 4A), but there was a complete ablation of F-actin levels immediately after stretch, and slow recovery to pre-stretch levels in 5 minutes (Figure 4B). The trajectory of traction recovery (30%–60%, Figure 1D) differed from that of stiffness (50%–90%, Figure 2 Untreated) and F-actin intensity recovery (50%–100%, Figure 3E,F), but these differences are likely attributable to slight differences in experimental protocols; tractions were measured in single isolated cells whereas OMTC and F-actin measurements were performed in confluent cell layers.

## Discussion

Dynamic changes in stiffness and traction forces in HBSM cells are consistent with our previous findings in human airway smooth muscle (HASM) cells and other adherent cell types [6], [7]. Because stretch-induced fluidization is virtually instantaneous, it would appear not to be mediated by signaling events, but rather to represent the immediate response of a fragile material to application of physical forces. Indeed, when we disrupted PI3K, Akt, Rho-kinase and MEK pathways, all of which are known to play key roles in the HBSM response to a prolonged stretch, there were no noticeable effects on the fluidization-resolidification response. We also found that transient stretch did not alter protein tyrosine phosphorylation profile (Figure 4A). In our previous study, when HASM cells were pretreated with phenylarsine oxide (PAO), which inhibits tyrosine phosphorylation, the recovery of traction forces following a transient stretch was largely ablated [6]. But in addition to its ability to inhibit tyrosine phosphatase activity, PAO also decreases cellular ATP content, and as such we cannot rule out the possibility that our previous results reflected the effect of ATP depletion, not phosphatase inhibition on recovery of traction forces. Indeed, we have shown previously that ATP depletion causes stiffness recovery to slow considerably [7]. Taken together, these results suggest that the extent of sudden fluidization depends primarily on the physical property of the fragile material, whereas the resolidification response is dependent upon ATP-driven events.

Like the mechanical changes (Figures 1 and 2), western blotting and actin staining showed that structural changes were prompt. The transient stretch-unstretch maneuver induced prompt disassembly followed by slow reassembly of F-actin filaments. These findings are consistent with those of Pender and McCulloch, who reported that F-actin in gingival fibroblasts is reduced by 50% at 10 s after stretch but increases more than 100% at 50 s after stretch [22]. Costa *et al* also found in human aortic endothelial cells there is rapid remodeling of stress fibers after mechanical perturbation [25].

Finally, we found that a transient stretch-unstretch maneuver caused cell fluidization whereas a transient compression-decompression of the same amplitude and timing did not (Figure 2 Compression). No changes in cell shape or the direction of cell alignment were observed, as has been reported for cells exposed to repetitive cycles of pure uniaxial compression [26]. Moreover, when cells are subjected to a stretch and hold maneuver, the traction forces and CSK stiffness become elevated and largely sustained, although followed by stress relaxation processes that are quite slow [7], [16]. Together, these findings suggest that the prompt destabilization of actin filaments and loss of traction forces are therefore localized to the unstretch phase of the stretch-unstretch manueuver, as if catch bonds [27] increasingly stabilize the CSK during the loading (stretch) phase, but upon unloading promptly release. Catch-like behavior of adhesion molecules and cell anchorage is well-established [27], but to our knowledge catch-like behavior of stress-bearing molecules within the CSK itself has never before been suggested. Importantly, catch-like behavior would also help to account for the unexplained but well-established observation that the CSK loss tangent (hysteresivity) systematically decreases as CSK tension increases [28], [29], indicating that the CSK becomes systematically more solid-like.

While molecular mechanism remains unclear, actin depolymerization responses we report here are certainly dissimilar from the fluidization responses observed in reconstituted actin-myosin networks, which are attributed to disruption of myosin crosslinks [17], [30], [31]. Similarly, we have reported that the perturbation of myosin crosslinks by stretch is a major factor in the fluidization of isolated airway smooth muscle cells and integrated tissues [32], [33]. However, disruption of myosin crosslinks alone fails to explain the underlying cytoskeletal plasticity [18], [34], its scale-free rheology [12], [35] or its universality in living cells [7]. Neither can myosin dynamics alone account for our previously reported differences between fluidization versus reinforcement observed in response to a homogenous versus an inhomogeneous stretch [6], or account for our current findings of rapid F-actin disassociation followed by a gradual recovery (Figure 3 and 4).

As such, we conclude that fluidization is caused not just by agitation or by relative motion, but rather by increased tensile forces that, upon sufficient loading, or perhaps upon release of that loading, can lead to dissociation of weak bonds or drive disassociation of actin with binding partners such as α-actinin or VASP, whose recruitment is facilitated by the force-dependent action of zyxin [36]–[38]. In that connection, zyxin is a logical candidate to account for the slow resolidification that is observed after unstretch, but could zyxin account for prompt fluidization during stretch? Increasing cytoskeletal tension causes the rate constant for zyxin dissociation (the off-rate) from focal adhesions to decrease [37] and mobilization of zyxin to stress fibers [38]. As such, during the “stretch” phase of the transient maneuver studied here, zyxin could not account for fluidization and, if anything, would act to reinforce stress fibers. On the other hand, during the unstretch phase of the transient maneuver, traction forces fall rapidly to zero [6]. Whether zyxin, during that low-tension phase, might demobilize from stress fibers sufficiently to account for actin disassembly is unclear and warrants investigation. Another plausible candidate is the actin severing protein, cofilin [39]–[41]. In its dephosphorylated form, cofilin binds and remodels actin filaments, increases its torsional and bending flexibility, and significantly lowers filament persistence length [42]–[44]. Taken together, these effects promote bending and disassociation of *in vitro* actin networks. How these effects mediate dynamics of stretch responses in intact cells remains unknown.

In summary, through cytoskeletal imaging and molecular probes we have elucidated a major role for F-actin dynamics in the acute fluidization-resolidification response, and discovered that prompt changes are mediated not through signaling pathways but are brought about by the direct action of tensile forces exerted on a fragile cell. For reasons that remain unclear, the depolymerization of the actin cytoskeleton is extremely rapid, and is localized to the unstretch phase of the maneuver.

## Materials and Methods

### Cell culture and pharmacological interventions

HBSM cells were isolated and propagated as previously described [45]. Cells were cultured in Dulbecco's modification of Eagle's medium (DMEM) supplemented with 10% fetal bovine serum (FBS), penicillin (100 U/ml) and streptomycin (100 µg/ml), and were placed in a humidified incubator at 37°C and 5% CO_2_. Cells between passage 3–7 were used for all experiments, and they were serum deprived for 24 h before being tested. For the traction force measurement, cells were seeded sparsely (∼2,000 cells/well) on type I collagen coated polyacrylamide gel substrates 4 h before experiment; for the OMTC measurements and actin staining, cells were seeded at ∼200,000 cells/well on gel substrates 24 h before experiment; for western blotting, cells were seeded at ∼200,000 cells/well in a 6-well plate on deformable membranes (type I collagen coated; Flexcell International Corporation). The following pharmacological interventions were used: LY294002 (inhibits the PI3K, 10µM), Triciribine (specifically inhibits the Akt/PKB, 10µM), SB203580 (inhibits p38 MAP kinase pathway, 10µM), PD098059 (inhibits the MEK, 30µM), Y27632 (inhibits Rho/Rho-kinase pathway, 10µM) and Latrunculin-A (inhibits actin polymerization and disrupts microfilament organization). The incubation time for all inhibitors was 20 min.

### Preparation of polyacrylamide substrates

Polyacrylamide gel substrates were prepared according to previous protocols [46]–[48]. The gels were made within 35 mm dishes (glass bottom, uncoated, P35-G-020-C; MaTek). The diameter and the depth of the gel substrates were 20 mm and 700 µm respectively. We adjusted the ratio between 40% acrylamide (Bio-Rad Laboratory) and 2% bis-acrylamide (Bio-Rad Laboratory) to produce the gel substrates with Young's modulus at 4 kPa because HBSM cells showed optimal proliferation rate on 4 kPa substrates (data not shown). After gel polymerization, the substrates were coated with 1.5 ml of type I collagen solution (0.1 mg/ml; Inamed Biomaterial) and stored overnight at 4°C. On the next day, the gels were washed with phosphate buffered saline, hydrated with 2 ml of DMEM supplemented with 0.5% FBS and stored in incubator at 37°C and 5% CO_2_.

### Stretch apparatus

We used a custom-built stretch setup [6]. A biaxial stretch was imposed by lowering a hollow circular punch indenter onto an elastic polyacrylamide gel. In response, cells within the indenter footprint were subjected to a uniform biaxial stretch. The stretch indenter was mounted on the objective of the microscope, coaxial to the objective lens. It was then lowered manually onto the underlying substrate by a pre-calibrated amount. To impose a 10% transient stretch, an indentation of 400 µm was imposed for 4 seconds. For transient tensile stretch (stretch-unstretch), we used a circular indenter with an inside diameter of 2 mm and an outside diameter of 3 mm; for transient compressive stretch (unstretch-restretch), we used a circular indenter with an inside diameter of 6 mm and an outside diameter of 7 mm [6].

### Traction force microscopy

Phase contrast images of the single HBSM cell and fluorescent images of microbeads embedded within the substrate were taken at different time points during the no-load baseline period, before the onset of the stretch, after stretch cessation and following cell detachment at the end of experiment. We used Fourier transform traction cytometry [24] to compute the cell traction forces. From the traction map, we obtained the net contractile moment, which is a scalar measure of the cell's net contractile strength. Nine single cells in 9 wells were used to repeat the traction force measurement.

### Optical magnetic twisting cytometry

The details of the OMTC technique are described elsewhere [12], [35]. First, we added the RGD-coated beads onto cells seeded on the gel substrates and waited for 20 min to allow the beads to bind to receptors on the cell surface. Next, we added inhibitors at the stated concentrations into each dish and waited for an additional 20 min. Soon after, we mounted the dish onto a microscope stage custom fitted with the bead twisting setup. The beads were magnetized horizontally and then twisted in an oscillatory magnetic field with a frequency of 0.75 Hz. From the ratio of the applied mechanical torque to the measured lateral bead displacement, we calculated the complex elastic modulus which is given by 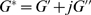, where 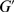 is the elastic modulus, or cell stiffness, which has units of Pascal per nanometer, 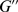 is the loss modulus which has units of Pascal per nanometer, and 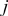 is the unit imaginary number −1. We measured 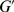 in 3–6 wells for each experimental condition. Data were reported as medians of the bead populations (244–709 beads on a hundreds of cells per experimental condition).

### Fluorescence staining of F-actin

Cells cultured on polyacrylamide gel dishes were fixed with 4% formaldehyde in PBS (for 10 min) at 1 s, 2.5 min or 5min after stretch. Fixed cells were then permeabilized with 0.1% triton X-100 in PBS for 10 min. F-actin filaments were then stained with Oregon Green 488 phalloidin (diluted 1∶40 in PBS; Molecular Probes). The stained cells were visualized with a 40× oil immersion lens equipped on the Nikon Eclipse TE300 microscope. Images were acquired and analyzed using the MetaVue software (Universal Imaging Corporation).

### Western blotting

HBSM cells were seeded on Flexcell plates with deformable membranes coated with type I collagen and subjected to 10% transient stretch of 4 s duration. Cells were harvested at 1 s, 2.5 min and 5 min after stretch cessation. Two wells were pooled for each condition. A non-stretch condition was also included as the negative control group. For phosphor-tyrosine blots, equal amounts of protein lysates from each condition were separated by SDS-PAGE, transferred onto nitrocellulose membranes and probed with 4G10 anti-phosphotyrosine antibody (Upstate/Millipore, cat# 05-321). For actin blots, equal volumes of the G-actin and F-actin fractions from each treatment condition were separated by SDS-PAGE, transferred to nitrocellulose membranes and probed with an actin antibody (Cytoskeleton, cat# AAN01).
